# Lymphocytic Choriomeningitis in Michigan

**DOI:** 10.3201/eid1205.050794

**Published:** 2006-05

**Authors:** Erik S. Foster, Kimberly A. Signs, David R. Marks, Hema Kapoor, Margaret Casey, Mary Grace Stobierski, Edward D Walker

**Affiliations:** * Michigan Department of Community Health, Lansing, Michigan, USA;; † United States Department of Agriculture, Okemos, Michigan, USA;; ‡ Michigan State University, East Lansing, Michigan, USA

**Keywords:** Lymphocytic Choriomeningitis, Arenavirus, Arenaviridae, Aseptic Meningitis, Serology, Mus musculus, Rodent-borne, Epidemiology, Electron microscopy, Pancreatitis

## Abstract

We summarize the first reported case of acquired lymphocytic choriomeningitis virus (LCMV) infection in Michigan to be investigated by public health authorities and provide evidence of the focal nature of LCMV infection in domestic rodents. Results of serologic and virologic testing in rodents contrasted, and negative serologic test results should be confirmed by tissue testing.

Lymphocytic choriomeningitis virus (LCMV) is a frequently unrecognized cause of aseptic meningitis and congenital infections in humans (*1**,**2*). First described in 1933, it is a rodentborne zoonosis associated with the common house mouse (*Mus musculus*) (*3*). Wild mice, often infected in utero, may not develop an effective immune response and remain asymptomatic carriers and shedders. Large-scale outbreaks of LCMV infection in humans have primarily been associated with contact with infected hamsters. Since 1960, 3 epidemics of LCMV infection involving at least 236 human cases have occurred in the United States; all were associated with Syrian hamsters as laboratory animals or pets (*4*). LCMV is shed in the urine, feces, saliva, milk, semen, and nasal secretions of chronically infected rodents. Routes of human exposure include aerosols, droplets, fomites, and direct contact with rodent excreta or blood (*3*). Recently, organ transplantation has been recognized as an additional mode of transmission for this virus (*5*). We describe the first reported case of meningitis due to LCMV infection in a Michigan resident.

## The Case

A 46-year-old woman previously in good health came to a community hospital emergency department on June 12, 2004, with a 1-week history of severe headache, body aches, photophobia, weakness, and fatigue. A viral syndrome was diagnosed on 2 previous physician visits. Prior medical history included migraine headaches. A complete blood count, blood culture, serum chemistry tests, chest radiograph, urinalysis, and lumbar puncture were performed. Abnormal results included the following: cerebrospinal fluid contained 520 leukocytes/mm^3^ with 100% lymphocytes, 19 erythrocytes/mm^3^, protein 128.6 mg/dL, and glucose 59 mg/dL. Serum glucose was 124 mg/dL; serum lipase level was elevated at 686 U/L. A computed tomographic (CT) scan of the brain without infusion showed no evidence of acute brain process; abdominal/pelvic scan showed inflammatory change adjacent to the tail of the pancreas, consistent with possible pancreatitis.

The patient was admitted to the hospital and placed in respiratory isolation with a diagnosis of acute meningitis, likely of viral origin, and mild pancreatitis. She was given supportive care with intravenous fluids, acyclovir, and pain medication.

After consultation with an infectious disease specialist, several diagnostic tests were performed, including serologic tests for adenovirus, *Chlamydophila psittaci*, antinuclear antibodies, cytomegalovirus, LCMV, coxsackie B virus types 1–6, and echovirus types 4, 7, 9, and 11; polymerase chain reaction (PCR) for herpes simplex virus 1 and 2; infectious mononucleosis screen; cryptococcal antigen testing; and urinary mumps antibody testing. Positive results included mumps antibody titer of immunoglobulin G (IgG) 2.66 (negative <0.91) and IgM 1.64 (negative <0.81), and LCMV immunofluorescence assay (IFA) titer of IgG 256 (negative <15) and IgM 320 (negative <20). Confirmatory testing at the Centers for Disease Control and Prevention (CDC) in Atlanta found the specimen IgG-reactive and negative for mumps by urinary antigen culture and PCR. The patient was born before widespread mumps vaccination; thus, results suggested a previous exposure. LCMV serologic testing by enzyme-linked immunosorbent assay (ELISA) showed an IgG titer of 1,600 (cutoff <100) and an IgM titer of 6,400, which indicated recent infection.

The patient improved and was released after 7 days of hospitalization. The family had owned 2 healthy pet rats for 2 years, although the patient had little direct contact with them. However, the patient reported that the family had been battling a severe rodent infestation for 6 months, since they no longer kept cats as pets. The family had been trapping 4–5 mice per night in the weeks before the patient's illness onset. No other family members reported illness.

Because of the substantial rodent infestation and continuing risk to others in the household, the Michigan Department of Community Health, together with the local health department and the US Department of Agriculture Wildlife Services, received permission from the patient to conduct a field study to determine the extent of infestation and prevalence of infection in mice and to provide counseling on health implications and control of the infestation.

## The Investigation

Following an initial site investigation, live traps were placed within the home and in the immediate area outdoors (within 10 m of the residence). Traps were visited daily for 2 days. All trapping and sampling procedures were performed according to CDC guidelines for sampling small mammals for virologic testing (*6**,**7*).

On July 28 and 29, 20 animals were captured, including 17 house mice, 1 white-footed mouse (*Peromyscus leucopus*), 1 short-tailed shrew (*Blarina brevicauda*), and 1 eastern chipmunk (*Tamias striatus*). Fecal pellets were also collected from the environment, traps, and pet rats' cage. From July 25 to 27, the homeowner caught 6 house mice in snap traps; the mice were frozen, and specimens were obtained.

During necropsy, blood samples were collected by saturation of Nobuto filter strips, and spleens were collected and frozen at -70°C. Spleen tissues and fecal pellets were homogenized, filtered, and inoculated into Vero cell cultures, which were maintained every 7 days with fresh maintenance media and observed daily for cytopathic effect. Serologic testing was performed on the Nobuto strips by CDC according to previously described methods (*8*). All cultures were screened by IFA staining with anti-LCMV mouse hyperimmune ascites fluid, obtained from CDC (lot #92-0038L) and diluted 1:800 in phosphate-buffered saline with 5% skim milk and 0.5% Tween 20 for 30 min at 37°C.

Twenty-two (96%) of 23 house mouse spleen tissue samples showed evidence of LCMV infection by virus isolation and IFA with specific LCMV antibodies. None of 14 fecal pellet suspensions showed evidence of LCMV by virus isolation or IFA. All Nobuto strips were negative for LCMV-specific antibodies by ELISA (Table). Confirmation PCR of a single virus isolate was conducted at the Special Pathogens Laboratories (CDC), and results were positive.

**Table:** Results of small mammal trapping and laboratory testing in a household exposure, July 25–29, 2004*

Species†	No. sampled	ELISA	Virus isolation‡ and IFA (%)	EM (%)
*Mus musculus*	23	0/23	22/23 (96)	5/5 (100)
*Peromyscus leucopus*	1	0/1	0/1	0/0
*Tamias striatus*	1	0/1	0/1	0/0
*Blarina brevicauda*	1	0/1	0/1	0/0

*LCMV, lymphocytic choriomeningitis virus; ELISA, enzyme-linked immunosorbent assay; IFA, immunofluorescence assay; EM, electron microscopy. †All species other than *M. musculus* were trapped outside the residence; 1 *M. musculus* was trapped outside, and the specimen was verified virus positive. ‡One isolate provided to the Centers for Disease Control and Prevention was confirmed LCMV-positive by polymerase chain reaction.

Five Vero cultures, positive by IFA, were observed by negative-stain electron microscopy grid preparation. This procedure confirmed virions consistent with an arenavirus in all specimens tested (Figure).

**Figure:**
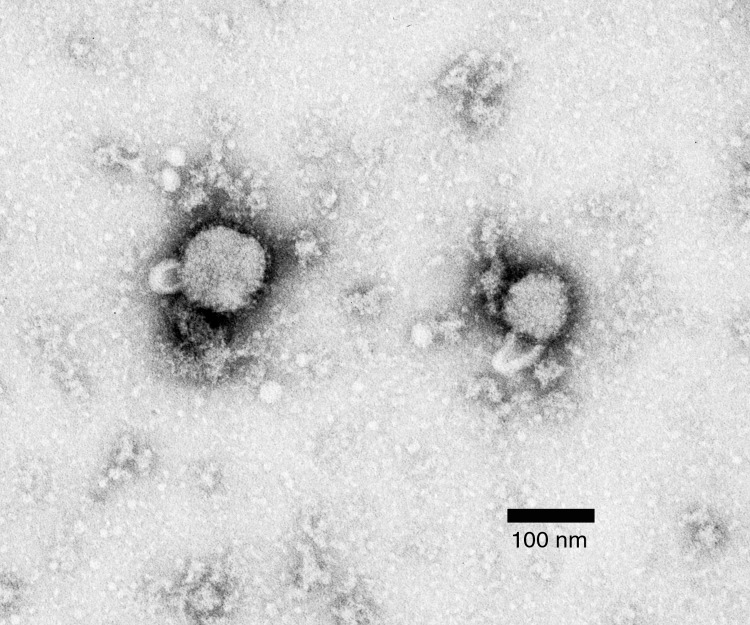
Methylamine tungstate negative-stain electron micrograph of arenavirus isolated from mouse spleen homogenate cultures that tested positive by immunofluorescence assay for lymphocytic choriomeningitis virus infection. Viral envelope spikes and projections are visible, and virion inclusions show a sandy appearance, indicating *Arenaviridae*.

## Conclusions

We describe the first documented case of acquired LCMV infection in Michigan. Evidence shows the highly focal nature of LCMV and the potential for human illness from exposure to the virus. Based on the patient's course of illness, dense rodent infestation in the patient's home, known routes of virus shedding, and mating and territorial ecology of the house mouse, we infer that the high infection rates in house mice caused her infection and subsequent illness. Investigators could not obtain samples from other residents of the house, so the household seroprevalence is undetermined.

Previous rodent serosurveys have shown focality, but few have provided evidence of such high infection rates in rodents. In this study, 96% of *M. musculus* examined were viremic. This result may be attributable to methods used to quantify infection status in the samples and the trapping intensity at a single focus. Infection rates of captured rodents may differ between rural and urban ecosystems, parks and housing complexes, and between housing complexes (*8*). Infection rates in natural populations have been estimated at 2.5% (California) and 21% (Washington, DC) (*9**,**10*). In urban Baltimore, however, single-dwelling units in the same neighborhood showed antibody prevalence to LCMV from 0% to 50% (*8*). In an LCMV epizootic of laboratory mice in the United Kingdom, bite transmission occurred and antibody prevalence was 67% in wild mice that were caught (*11*). Over a few generations, every member of a colony may become infected, as vertical transmission approaches 100% efficiency (*12*).

As was demonstrated by our results and suggested in earlier research, serologic testing of rodents underestimated overall infection rate (*8*), possibly because circulating antibodies were lacking in vertically infected mice. Oldstone and Dixon found that in the offspring of infected mice, antibodies to LCMV were sequestered in the kidneys and undetectable in blood (*13*). In our study, results of serologic testing on Nobuto strips were negative for all specimens, while results of virus isolation and IFA from spleen homogenates were positive for LCMV in 96% of *M. musculus* that were sampled and in 85% of all animals tested. Thus, negative serologic test results in rodents should be confirmed by tissue testing, such as virus isolation and IFA, or other methods, such as PCR. While most house mice were infected, none of the fecal pellets collected from traps were positive by virus isolation. This finding may be due to the fragile nature of LCMV in the environment or the predilection of the virus for rodent kidneys (*14*).

LCMV most likely represents an underdiagnosed, endemic zoonotic disease. Future goals include public health surveillance enhancements, physician education, and epidemiologic studies. Surveillance can be improved by adding LCMV to reportable disease lists and including a question about rodent exposure on case report forms for aseptic meningitis. Improved surveillance data can be used to educate clinicians on the range of illnesses caused by LCMV and the potential for acquired and congenital infection by exposure to rodents; this increased awareness would increase diagnostic testing and case identification. Improved case identification could lead to future studies to determine potential environmental, social, and economic risk factors, which would allow prevention and control efforts to be focused on vulnerable populations.
